# Predictors of Vaccine Hesitancy among Health Care Workers during the COVID-19 Pandemic

**DOI:** 10.3390/ijerph19127123

**Published:** 2022-06-10

**Authors:** Timothy R. Elliott, Paul B. Perrin, Mark B. Powers, Katelin S. Jacobi, Ann Marie Warren

**Affiliations:** 1 Department of Educational Psychology, Texas A&M University, College Station, TX 77843, USA; katelinj12@tamu.edu; 2 Department of Psychology, Virginia Commonwealth University, Richmond, VA 23284, USA; pperrin@vcu.edu; 3 Trauma Research Consortium, Baylor Scott & White Research Institute, Department of Trauma, Critical Care and Acute Care Surgery, Baylor University Medical Center, Dallas, TX 75246, USA; mark.powers1@bswhealth.org (M.B.P.); annmarie.warren@bswhealth.org (A.M.W.)

**Keywords:** COVID-19, SARS-CoV-2, health care providers, vaccine hesitancy

## Abstract

Most studies of COVID-19 vaccine hesitancy among health care workers (HCWs) have been descriptive, few have tested models to predict hesitancy, and none have examined the possible relationship between HCWs’ distress and vaccine hesitancy. This study examined predictors of COVID-19 vaccine hesitancy, including HCWs’ distress after taking into account HCW sex, doctoral-level status, race, age, and exposure to COVID-19. Further, it examined specific reasons HCWs endorsed for their hesitancy. 266 HCWs in the United States (U.S.). completed an online survey administered in January 2021, following the availability of the vaccine for HCWs in the U.S. The survey assessed demographics, depression, anxiety, COVID-19 vaccine hesitancy, and reasons for hesitancy. A comprehensive linear regression model explained 72.2% of the variance in COVID-19 vaccine hesitancy. HCWs were more hesitant if they did not know someone personally who had tested positive. Distress had no effect. The reasons most predicting vaccine hesitancy included safety, potential side effects, believing the risks from COVID-19 were lower than from the vaccine, not feeling at risk for getting COVID-19, and current pregnancy. Rather than rely on providing information about the COVID-19 vaccines to HCWs, strategies that address their concerns are required to promote vaccine acceptance. Contemporary issues of political polarization, misinformation and mistrust are likely to contribute to the concerns HCWs have about the COVID-19 vaccines.

## 1. Introduction

The novel coronavirus disease 2019 (COVID-19) has been described as the first occupational disease of this century, primarily because of the disproportionate risk of exposure, infection and distress among healthcare personnel across the globe [1,2]. Throughout the pandemic, a significant percentage of healthcare workers (HCWs) report clinical levels of depression (rates ranging from 5% to 51%) and anxiety (14.5% to 44.6%) [3,4,5]. Those who have routine and direct patient contact (e.g., nurses, first-responders) appear to be more likely to report psychological distress (i.e., elevated symptoms of depression and anxiety) than physicians and other doctoral-level providers [4,6]. The majority of HCWs are women [7], and there is evidence that they may be at a higher risk for distress than men during the pandemic [4,6].

Unfortunately, the HCWs at risk for distress during the pandemic are also more likely to report vaccine hesitancy [8,9]. HCWs who are hesitant about the COVID-19 vaccines express concerns about a lack of information about the vaccine, its efficacy, and potential side effects [10]. Physicians and nurses are considered the most trusted sources for vaccine information, and they effectively promote vaccine acceptance among patients of all ages [11]. However, a study of HCWs in a pediatric hospital found nurses were significantly less likely than physicians to recommend COVID-19 vaccines to patients [12].

Most studies of COVID-19 vaccine hesitancy among HCWs are descriptive, few have tested models to predict hesitancy, and to the best of our knowledge few have examined the possible relationship between HCWs’ distress and vaccine hesitancy. Identifying predictors of vaccine hesitancy among HCWs could potentially inform the development of appropriate policies and services to address these factors. Unfortunately, the few studies of HCW distress and vaccine hesitancy have been compromised by poor instrumentation [13] and contradictory results. For example, a study of Polish HCWs found depressive symptoms in the previous week predicted a greater willingness to receive a COVID-19 vaccine [14], but the opposite relationship was found among Polish medical students [15]. Significant levels of depression and anxiety are associated with vaccine hesitancy in a community survey using well-validated measures [16], and those who have been diagnosed with depressive and anxiety disorders are also hesitant to receive a COVID-19 vaccine [17].

Depression and anxiety are associated with worry, passivity, wishful thinking, and avoidant tendencies [18]. Consequently, individuals with these issues are less likely to engage in proactive, self-care behaviors. Individuals who endorse symptoms of depression appear susceptible to misinformation about COVID-19 vaccines [19], and a recent review of the relevant research to date found fear and anxiety were associated with COVID-19 hesitancy [20].

We conducted the present study to examine predictors of COVID-19 vaccine hesitancy, including HCWs’ depression and anxiety after taking into account HCW sex, doctoral-level status, race, age, and exposure to COVID-19. Further, we examined specific reasons HCWs endorsed for their hesitancy at the final step of our model. In this manner, we could determine if individual reasons would account for any variance in vaccine hesitancy above and beyond any variance attributable to distress. This approach could potentially provide us with information about the psychological characteristics that might influence vaccine hesitancy, and in the process, inform efforts to address HCWs hesitancy in ways that might facilitate their willingness to take the vaccine and recommend it to others.

## 2. Materials and Methods

### 2.1. Study Design

Data were collected in a cross-sectional survey of adults (18 years of age and older) living in the United States (including Puerto Rico). The study was approved by the Baylor Scott & White Institute’s Institutional Review Board (IRB; #020-235). These data are part of a larger, longitudinal project that began in June and July 2020. Participants were recruited using the Qualtrics^TM^ survey platform to obtain a U.S. representative sample. To participate, individuals had to be at least age 18, and have mastery of the English language. Qualtics redacted participants’ names and email addresses in correspondence with the research team. Qualtrics assigned response identification numbers to participants that allowed the research team to link survey responses across measurement occasions while ensuring participant anonymity.

Of the 5023 individuals who completed the first administration hosted by the Qualtrics^TM^ survey platform, 1419 self-identified as HCWs. Qualtrics sent a subsequent survey to all participants who completed the first survey. Data for this study were culled from the third survey that was distributed from 4 January to 7 January 2021. COVID-19 vaccines were first available to HCWs in the U.S. in December 2020. An item was added in the following month in the third survey to assess vaccine hesitancy among participants. 266 HCWs completed this third survey.

The Qualtrics^TM^ “speed check” validation criteria were used to delete participant surveys that were completed at an implausible rate (i.e., under half the median response time). Data from individuals who did not complete the survey, who responded with nonsensical words to open-ended items, and who provided inconsistent answers or provided “straight-line” responses were also discarded. Further method details have been previously published [21].

HCWs self-identified on items concerning respondent occupation, and this group included physicians, nurses, pharmacists, occupational therapists, optometrists, speech pathologists, physical therapists, nursing assistants, social workers, home health workers, and chiropractors. Doctoral status was determined by responses to items concerning occupation status as a general practitioner, surgeon, dermatologist, dentist, psychologist, pediatrician, optometrist, orthodontist or other doctoral-level provider. Non-doctoral level providers self-identified as a nurse (“LPN, RN, NP, Assistant, etc.” and “Other healthcare professional (laboratory, housekeeping, medical records, nutritionist, social worker, hospice, etc.”).

### 2.2. Study Measures

Participants reported demographic information that was used in this study (age, highest educational degree, race/ethnicity, occupational status, sex). The Patient Health Questionnaire-8 (PHQ-8) and the Generalized Anxiety Disorder Scale–7 (GAD-7) were used to assess symptoms of depression and anxiety, respectively [22]. The PHQ-8 has eight items and the GAD-7 has seven items that are rated on a 0 (“not at all”) to 3 (“nearly every day”) Likert-type scale. Higher total scores indicate greater endorsement and severity of symptoms, and these were used in our analyses. On both instruments a score of 10 indicates a moderate level of severity, and a score of 15 indicates severe symptomology. Both instruments are suitable for use in community research and demonstrate respectable psychometric properties [22]. A single item was used to assess self-reported burnout: “Have you felt more or less burnout since the COVID-19 outbreak?” For use in our analyses, responses were coded as 1 (I have felt less burnout), 2 (I have not felt more or less burnout), or 3 (I have felt more burnout). The total score was used for analyses.

Respondents were asked to report their personal exposure to COVID-19: (1) did the respondent personally test positive for COVID-19 or (2) know someone who has tested positive. Vaccine hesitancy was assessed with a single item: “If a COVID-19 vaccine were available today in sufficient supply to vaccinate everyone who wants to receive it, would you take the vaccine?” Response options included “yes,” “no,” or “maybe.” If respondents answered either “no” or “maybe,” they were presented with the following possible reasons for their hesitancy: “I have concerns regarding safety, I have concerns regarding potential side effects, I want to wait a few weeks or months until others have taken it, I have concerns regarding how effective the vaccine is, I have religious reasons, I don’t feel at risk for getting COVID-19, I believe the risks from the COVID-19 infection are lower than from the vaccine and I am currently pregnant.” Respondents rated each reason as 0 (not selected) or 1 (selected). Participants were provided with an open-ended option to list their own reasons for vaccine hesitancy (prompted with stem, “Other”). Only seven participants responded to this prompt, and each participant listed only a single reason. These responses were idiosyncratic (e.g., “never had a flu shot & very healthy,” “awaiting guidance from my physician,” “already have immunity”), and could not be reasonably included with the existing options. Therefore, these seven responses were not used in subsequent analyses.

## 3. Analytic Plan

All analyses were conducted using IBM SPSS Statistics 28. Descriptives were calculated for the sample in terms of demographics, COVID-19 vaccine hesitancy level, distress, and reasons for vaccine hesitancy. A hierarchical linear regression was computed with COVID-19 vaccine hesitancy as the outcome variable (responses to If a COVID-19 vaccine were available today in sufficient supply to vaccinate everyone who wants to receive it, would you take the vaccine? 1 = Yes, 2 = Maybe, 3 = No). Demographics (age, race [1 = White, 0 = Non-White], biological sex (1 = female, 0 = male), and advanced degree status (1 = advanced degree, 0 = bachelor’s or lower) were included as Step 1 predictors; having tested positive previously for COVID-19 (1 = Yes, 0 = No) and knowing someone who has tested positive previously for COVID-19 (1 = Yes, 0 = No) as Step 2 predictors; burnout, anxiety, and depression as Step 3 predictors; and eight possible reasons why participants might not receive the COVID-19 vaccine (1 = reason selected, 0 = reason not selected) as a block at Step 4. Because the outcome of vaccine hesitancy had three progressive levels (1 = Yes, 2 = Maybe, 3 = No) such that higher scores reflected greater vaccine hesitancy, a linear regression was most appropriate. We were not interested simply in whether participants would receive the vaccine, but also if they were in the middle “maybe” level. Those predictors in the model with larger standardized β weights would be those that most account for unique variance in vaccine hesitancy.

## 4. Results

### 4.1. Descriptive Statistics

Descriptive statistics regarding participants’ demographics and COVID-19 vaccine hesitancy levels appear in Table 1. Nearly three quarters of participants responded “yes” to wanting to get the COVID-19 vaccine, though a substantial portion of the remaining participants responded “no.” Participants’ average distress scores were: depression (PHQ-8 *M* = 3.56, *SD* = 4.60), anxiety (GAD-7 *M* = 3.27, *SD* = 4.46), and burnout (*M* = 2.00, *SD* = 0.98). The average scores on the PHQ-8 and GAD-7 are similar to scores observed on these instruments in studies of HCWs, essential workers, and the general population during the COVID-19 pandemic [5,21].

### 4.2. Multiple Regression

The stepwise multiple regression results appear in Table 2. Step 1 was statistically significant, *F*(4, 259) = 6.59, *R*^2^ = 0.092, *p* < 0.001. Higher vaccine hesitancy was significantly and uniquely associated with female sex (β = 0.13, *p* = 0.049) and not having an advanced degree (β = −0.21, *p* = 0.001) but not the other predictors. With the addition of the COVID-19 variables as predictors in Step 2, the overall model was still statistically significant, *F*(6, 257) = 6.74, *R*^2^ = 0.136, *p* < 0.001. The model was a significant improvement over the previous model, accounting for an additional 4% of the variance in vaccine hesitancy (*R*^2^*_inc_ =* 0.044, *p* = 0.002). Within this model, female sex was no longer statistically significant, though advanced degree status remained significant, and knowing someone who had tested positive was associated with lower vaccine hesitancy (β = −0.20, *p* = 0.001). With the addition of the distress variables as predictors in Step 3, the overall model remained statistically significant, *F*(9, 254) = 4.72, *R*^2^ = 0.143, *p* < 0.001, but it was not a significant improvement over Model 2 (*p* = 0.547). Contrary to our expectations, none of the distress variables were associated with vaccine hesitancy.

With the addition of the possible reasons why participants might not receive the COVID-19 vaccine as predictors in Step 4, the overall model remained statistically significant, *F*(8, 246) = 37.52, *R*^2^ = 0.722, *p* < 0.001. This step accounted for an additional 57.9% of variance in vaccine hesitancy (*R*^2^*_inc_ =* 0.579, *p* < 0.001). Within this final model, knowing someone who tested positive was still associated with lower vaccine hesitancy. White race appeared uniquely associated with being less vaccine hesitant. However, White race was not a significant predictor in the first three steps of the hierarchal linear regression. Although it met a statistically significant level in the fourth step of the equation, this significance is likely spurious and simply error due to a statistical suppressor effect. As a result, it will not be treated as meaningful or interpreted further. Out of the possible reasons for not obtaining the COVID-19 vaccine, having concerns regarding safety (β = 0.40, *p* < 0.001), having concerns regarding potential side effects (β = 0.28, *p* < 0.001), and believing the risks from COVID-19 were lower than from the vaccine (β = 0.22, *p* < 0.001) were most strongly associated with increased vaccine hesitancy. These first two concerns were those most commonly endorsed by participants (Table 2), followed by wanting to wait a few weeks or months until others have taken it and concerns about how effective the vaccine is. Other reasons that contributed significantly at the step included not feeling at risk for getting COVID-19 (β = 0.18, *p* < 0.001) and current pregnancy (β = 0.14, *p* < 0.001); however, as depicted in Table 2, very few participants endorsed these two concerns.

## 5. Discussion

Vaccine hesitancy among HCWs during this time is an alarming issue because this behavior may facilitate transmission of the virus and jeopardize patient, co-worker and their own personal health, and it may promote vaccine hesitancy among others [11,23]. The stress and strain of the COVID-19 pandemic on HCWs is well-documented. Yet our results revealed no meaningful relationship between HCW distress and vaccine hesitancy, despite our expectation that a relationship would exist. Our predictive model found personal reasons for vaccine hesitancy accounted for a considerable amount of variance at the final step of the equation, above and beyond variance attributed to HCW sex, advanced degree status and prior experience with a person who had a positive COVID-19 diagnosis.

The percentage of HCWs who reported hesitancy in our sample (17% = no, 9% = maybe) slightly exceeds the average percentage of HCWs who are hesitant (22.51%, based on a review of studies that included a total of 76,471 participants) [9]. Although previous research reports differences in vaccine hesitancy as a function of gender, race/ethnicity, and HCW occupational status, our predictive model found that specific, individual-level concerns had strong and unique associations with hesitancy independent of these demographic characteristics. Consistent with the extant literature, HCWs who were hesitant in the current study were chiefly concerned with the safety, efficacy and potential side effects of the COVID-19 vaccines [9].

To a great extent, vaccine hesitancy among HCWs has been addressed as a matter of system and institutional policy. Health care delivery systems and professional organizations in health care services have ethical and fiduciary obligations to protect the health and well-being of patients and their families, and that of their colleagues and co-workers [24,25]. For health care professionals, vaccines are typically construed as part of a professional duty to “do no harm”; for some health care professionals, COVID-19 vaccines represent an ethical, if not moral, obligation of beneficence and fidelity, recognizing the need for and actively promoting the common good (e.g., the American Psychological Association’s ethical code of conduct) [26]. Nevertheless, HCWs who doubt the safety of the vaccines and worry whether it could harm their patients may believe it is their ethical responsibility to protect their patients from potential harm.

Our results indicated that a significant segment of HCWs harbored concerns about COVID-19 vaccines, and imply that these should be addressed in order to facilitate vaccine acceptance among these individuals. We cannot assume that all HCWs receive adequate information about the development, safety and potential side effects of these vaccines [27]. Moreover, we cannot assume that information alone will offset the mistrust, misinformation and motivated reasoning that exists among polarized factions throughout our communities and evident among HCWs [27,28]. Policies that mandate vaccines may inadvertently reinforce mistrust, perceived inequities in the workplace and, in absence of legitimate efforts to address questions about the vaccine, an unjust disregard of their concerns and their status [24,27,29].

Rather than rely on the provision of information, experts in persuasion and group dynamics recommend strategies that recognize the specific concerns maintained by individuals, and the subjective group norms that influence their behavior, and consider strategies that appeal to their professional obligations and their moral and ethical integrity to patients and families [28,29]. This requires working with individuals who are seen as credible and respected in these groups, who will recognize and address their concerns in ways that might facilitate more prosocial norms amenable to vaccine acceptance [27,28,29]. Evidence-based practices for promoting COVID-19 vaccine acceptance recommend coordinated efforts at the organizational and individual (e.g., HCWs, patients, families) levels to maximize effectiveness [30]. Although several of these strategies reflect and expand upon some of the observations we describe (e.g., accurate information, prosocial behavior), we believe some of the recommendations made for patient education materials also apply to HCWs. For example, our findings imply that promoting vaccine acceptance among HCWs might benefit from efforts to prepare HCWs for misinformation and conspiracy theories, training them with strategies to detect weak, misleading arguments that ignore scientific approaches and available empirical data [28]. This kind of intellectual “inoculation” can effectively reduce susceptibility to misinformation and “fake news” [28], and can be integrated into fact-based, conversational, and positively-framed interactions with patients and families [30]. These strategies may help HCWs and patients to focus on the safety of the vaccines, and perhaps see how the benefits of the vaccines and low likelihood of side effects are preferable to the greater and well-documented risks associated with COVID-19.

There are several important limitations to this study. Our sample is limited to those HCWs from the larger sample who were willing to complete the third survey from which these data were culled. Participants were asked about vaccine hesitancy in the first months of its availability in the U.S., and may not reflect their willingness in the following months during which they would likely have had interactions with co-workers who had received the vaccine and experienced no ill effects. More established measures are now available for measuring vaccine hesitancy; the current study relied on an item that was developed for this particular survey. The cross-sectional design and the low response rate (from the first to the third survey) limit the generalizability of the study. Further, our survey required participants to respond to specific reasons for their hesitancy, and offered a limited opportunity for participants to list their own reasons for their hesitancy (in response to the open-ended “other” option). This feature of our survey likely imposed a forced choice bias insensitive to the broad range of personal reasons participants may have for their vaccine hesitancy. Qualitative methods may be required to understand and appreciate the many reasons why HCWs are hesitant to receive a COVID-19 vaccine.

## 6. Conclusions

Our study indicated that individual reasons for COVID-19 vaccine hesitancy among HCWs were not associated with their level of distress, and these reasons accounted for more variance in vaccine hesitancy than other factors previously deemed important in previous research (e.g., gender, race/ethnicity, advanced degree). The percentage of HCWs who reported vaccine hesitancy in our study is consistent with the average rate observed in the extant literature. The final model accounted for 72.2% of variance in vaccine hesitancy. The individual reasons most strongly associated with vaccine hesitancy included concerns about safety, concerns about side effects, and believing the risks from COVID-19 were lower than from the vaccine. Political arguments and polarization, religious beliefs, mistrust of science and expertise, misinformation, and conspiracy theories have been associated with vaccine hesitancy throughout modern history [11], and these issues are associated with COVID-19 vaccine hesitancy among people in general [31,32]. Rather than rely solely on providing accurate, available information about the COVID-19 vaccines to HCWs, it may be important to recognize that these same issues that contribute to vaccine hesitancy throughout our communities are also associated with vaccine concerns among HCWs [27,28]. HCWs are respected and influential sources of vaccine information, but their hesitancy to obtain a COVID-19 vaccine jeopardizes their personal health, and increases the risk of transmission to patients, co-workers and, by extension, to their constituent family members. It is vitally important that health care systems identify and implement programs that effectively address and allay HCW concerns about COVID-19 vaccines to facilitate their acceptance.

## Figures and Tables

**Table 1:** Participant (*n* = 266) Demographics and COVID-19 Vaccine Hesitancy Level.

Variable	*M* or *N*	*SD* or %
Age, *M*, *SD*	53.34	12.50
Race/Ethnicity, *N*, %		
White	219	82.3
Asian	26	9.8
Hispanic	7	2.6
Black	10	3.8
Other	4	1.5
Sex, *N*, %		
Male	90	33.8
Female	176	66.2
Education Level, *N*, %		
Non-Doctoral	150	56.4
Doctoral	116	43.6
Had Tested Positive for COVID-19, *N*, %	32	12.0
Knew Someone Who Tested Positive for COVID-19, *N*, %	230	86.5
Would you get the COVID-19 vaccine?, *N*, %		
Yes	196	73.7
Maybe	24	9.0
No	46	17.3

**Table 2:** Regression Results and Percentages of Participants Reporting Vaccine Concerns.

Model	Predictor	Beta	*p*-Value	% Endorsed
1	Age	−0.11	0.095	-
	White Race	−0.03	0.665	-
	Female Sex	0.13	0.046	-
	Advanced Degree	−0.21	0.001	-
2	Age	−0.12	0.060	-
	White Race	0.00	0.950	-
	Female Sex	0.11	0.070	-
	Advanced Degree	−0.20	0.001	-
	Tested COVID Positive	0.08	0.164	-
	Know Someone Tested Positive	−0.20	0.001	-
3	Age	−0.12	0.062	-
	White Race	−0.01	0.900	-
	Female Sex	0.11	0.076	-
	Advanced Degree	−0.19	0.002	-
	Tested COVID Positive	0.07	0.275	-
	Know Someone Tested Positive	−0.19	0.001	-
	Burnout	−0.06	0.341	-
	Anxiety	0.10	0.274	-
	Depression	−0.02	0.802	-
4	Age	0.01	0.883	-
	White Race	−0.07	0.047	-
	Female Sex	0.04	0.282	-
	Advanced Degree	−0.04	0.304	-
	Tested COVID Positive	0.06	0.115	-
	Know Someone Tested Positive	−0.11	0.003	-
	Burnout	0.03	0.440	-
	Anxiety	0.05	0.383	-
	Depression	−0.04	0.499	-
	I have concerns regarding safety	0.40	0.000	16.5
	I have concerns regarding potential side effects	0.28	0.000	17.7
	I want to wait a few weeks or months until others have taken it	0.08	0.091	10.5
	I have concerns regarding how effective the vaccine is	0.02	0.661	9.0
	I have religious reasons	0.02	0.628	0.8
	I’m currently pregnant	0.14	0.000	0.4
	I don’t feel at risk for getting COVID-19	0.18	0.000	0.8
	I believe the risks from the COVID-19 infection are lower than from the vaccine	0.22	0.000	4.1

## Data Availability

Data are available from the corresponding author upon request.
